# Efficacy of combined angiotensin II receptor blocker with tripterygium glycosides on diabetic nephropathy: A protocol for meta-analysis

**DOI:** 10.1097/MD.0000000000025991

**Published:** 2021-06-04

**Authors:** Chang-e Ma, Pei Yu, Wei Wei, Xiao-qin Chen

**Affiliations:** a Department of Endocrinology; b Department of Nephrology; c School of Nursing, Medical College of Hexi University; d Department of Clinical Laboratory, Zhang Ye People's Hospital Affiliated to Hexi University, Zhangye City, Gansu, China.

**Keywords:** angiotensin II receptor blockers, diabetic nephropathy, tripterygium glycoside

## Abstract

**Background:**

Several studies have reported good results for angiotensin II receptor blockers (ARB) combined with tripterygium glycosides (TGs) in the treatment of diabetic nephropathy (DN). However, because a small number of cases were included in each study, the statistical power was limited. Therefore, we performed a protocol for meta-analysis to further evaluate the clinical efficacy and safety of combined ARB and TGs in treatment of DN.

**Methods:**

The protocol was written following the Preferred Reporting Items for Systematic Reviews and Meta-Analyses Protocols (PRISMA-P) statement guidelines. We searched PubMed, Embase, Cochrane Central Register of Controlled Trials, Web of Science, China National Knowledge Infrastructure (CNKI), Wanfang Data, Science Direct up to April 2021. Outcome measures were 24-h urinary total protein, urinary albumin excretion rate, serum creatinine, blood urea nitrogen, albumin, hemoglobin A1c, β2-microglobulin and serum glutamic pyruvic transaminase. The risk of bias assessment of the included studies was performed by two authors independently using the tool recommended in the Cochrane Handbook for Systematic Reviews of Interventions (version 5.1.0). We performed meta-analysis using STATA 11.0.

**Results:**

The review will add to the existing literature by showing compelling evidence and improved guidance in clinic settings.

**Conclusion:**

The findings will provide helpful evidence for the application of combined ARB and TGs in the treatment of DN.

**OSF registration number:**

10.17605/OSF.IO/ARGE3

## 1 Introduction

Diabetic nephropathy (DN) is a microvascular complication of diabetes concerning the glomerulus, renal tubules, and other renal structures, and is the main cause of end-stage renal failure (ESRF).^[1,2]^ With the incidence of diabetes increasing in recent years, the number of cases of DN has also increased. The National Diabetes Alliance estimates that, by 2030, there will be about 552 million people with diabetes globally, and nearly one-third of them will have DN.^[3]^ However, the pathogenesis of diabetes is still not clear and there are currently no effective treatments to prevent the progression to ESRD.^[4]^

Published studies have confirmed that persistent proteinuria was an independent risk factor for the progression of DN and that it was also one of the independent important prognostic factors.^[5,6]^ Therefore, reducing persistent proteinuria has become an important treatment purpose. Many clinical trials have confirmed that the renin angiotensin system and angiotensin II receptor blockers (ARB) can reduce the levels of urinary protein in patients with DN, thereby delaying its progression. However, only use of ARB for treatment of DN was not ideal.^[7,8]^

Tripterygium glycoside (TG) is a Chinese patent medicine and active compound extracted from the roots of a Chinese herb named *Tripterygium wilfordii*.^[9]^ Functions like anti-inflammation and immunosuppression of TG have been well proved and has been widely used for the treatment of various autoimmune and inflammatory diseases.^[10]^ The immunosuppressive action of TG has been generally attributed to its suppression of T or B-lymphocyte functions, including T-cell or B-cell apoptosis induction, as well as inhibition of lymphocyte proliferation.^[11]^

Several studies have reported good results for ARB combined with TGs in the treatment of DN. However, because a small number of cases were included in each study, the statistical power was limited. Therefore, we performed a protocol for meta-analysis to further evaluate the clinical efficacy and safety of combined ARB and TGs in treatment of DN.

## 2 Materials and methods

### 2.1 Protocol registration

The prospective registration has been approved by the Open Science Framework registries, and the registration number is 10.17605/OSF.IO/ARGE3. The protocol was written following the Preferred Reporting Items for Systematic Reviews and Meta-Analyses Protocols (PRISMA-P) statement guidelines.^[12]^ Ethical approval and patient consent are not required because this study is a literature-based study.

### 2.2 Searching strategy

We searched PubMed, Embase, Cochrane Central Register of Controlled Trials, Web of Science, China National Knowledge Infrastructure (CNKI), Wanfang Data, Science Direct up to April 2021. The following keywords and medical subject headings were searched: diabetic nephropathies, diabetic nephropathy, diabetic kidney disease, diabetic kidney diseases, DN and TGs. The first author will conduct a preliminary screening based on the title to eliminate any research not related to the topic. Subsequently, all remaining abstracts will be reviewed by the primary author, and the selection criteria are applied. Studies identified for full text review will be evaluated by 2 authors for inclusion in the study. Disagreements will be resolved through a discussion with a third review author. A manual search of the bibliographies of included studies is performed to ensure that the overall search was comprehensive and complete.

### 2.3 Inclusion and exclusion criteria

Inclusion criteria:

1. Research type: randomized controlled trials.
2. Research subjects: patients were clinically diagnosed with DN.
3. Interventions: The control group was treated with ARB and the experimental group was added with TG on the basis of the control group.
4. Outcome measures: 24-h urinary total protein, urinary albumin excretion rate, serum creatinine, blood urea nitrogen, albumin, hemoglobin A1c, β2-microglobulin and serum glutamic pyruvic transaminase.

Exclusion criteria:

1. Non-randomized controlled or semi-randomized controlled trials.
2. Other specific types of diabetes other than type 1 diabetes mellitus (T1DM) and type 2 diabetes mellitus (T2DM).
3. Kidney damage resulting from diseases other than T1DM or T2DM.
4. Other Chinese medicines being used in the control group or the experimental group.
5. Lack of rigorous experimental design, inappropriate statistical method or lack of related outcome measures.

### 2.4 Data extraction

Two independent authors will extract the following descriptive raw information from the selected studies: study characteristics such as author, study design, study language, publication year, mean follow-up period; patient demographic details such as number, average age, body mass index and gender ratio; details of interventions, and outcome measures. If the data is missing or unable to be extracted directly, we will contact the corresponding authors to ensure that the information integrated. Otherwise, we will calculate them with the guideline of Cochrane Handbook for Systematic Reviews of Interventions 5.1.0. If necessary, we will abandon the extraction of incomplete data.

### 2.5 Quality assessment

The risk of bias assessment of the included studies was performed by two authors independently using the tool recommended in the Cochrane Handbook for Systematic Reviews of Interventions (version 5.1.0). This tool included seven aspects which were sequence generation (selection bias), allocation sequence concealment (selection bias), blinding of participants and personnel (performance bias), blinding of outcome assessment (detection bias), incomplete outcome data (attrition bias), selective outcome reporting (reporting bias) and other bias (baseline balance and fund). Additionally, each of the aspects was ranked low risk of bias, high risk of bias, and unclear risk of bias.

### 2.6 Statistical analysis

We will perform meta-analysis using STATA 11.0 (http://www.stata.com; Stata Corporation, College Station, TX). The Q-test and I^2^ values will be used to indicate inter-study heterogeneity. When the *P*-value of Q-test > .1 and I^2^ < 50%, a fixed-effects model was applied; otherwise, a random-effects model was used. Binary variables were expressed by odds ratio with 95% confidence interval, and continuous variables by mean difference with 95% confidence interval. If significant heterogeneity is found, we will try to explore the source of heterogeneity by subgroup analysis based on specified effect modifiers as follows: interventions, publication year, participant's average age, sample size, publication language, and so on.

## 3 Discussion

There are still no effective treatments for DN or ESRD. ARB can improve proteinuria both in early and late stages of type 2 DN and alleviate the progression of kidney injury.^[13,14]^ TG is widely used to treat primary glomerulonephritis and immune-related nephritis. In DN, TG not only improved proteinuria, but also alleviated kidney pathological changes and reduced the inflammation levels of kidney via p38 mapk pathway. In order to research the effect of combined TG and ARB on DN, we performed this meta-analysis.

Traditional Chinese medicine (TCM) has a long history of medicinal use and the side effects of TCM are relatively minor. The combination of TCM and Western medicine could prove to be an important method for treating DN. This meta-analysis provides a new perspective in regard to the combined treatment using TG and ARB on DN. For better understanding and optimizing TG combined with ARB in DN, we need to standardize clinical trials and analyze larger multicenter randomized controlled trials.

## Author contributions

**Conceptualization:** Pei Yu.

**Data curation:** Pei Yu.

**Funding acquisition:** Wei Wei.

**Investigation:** Wei Wei.

**Writing – original draft:** Chang-e Ma.

**Writing – review & editing:** Xiao-qin Chen.
